# Surgery for Superior Cerebellar Artery Aneurysm

**DOI:** 10.21315/mjms-05-2025-348

**Published:** 2025-12-31

**Authors:** Sarah Atiqah Mohd Zamri, Nurfaten Hamzah, Thinesh Kumaran Jayaraman

**Affiliations:** 1 Department of Neurosurgery, Tunku Abdul Rahman Neuroscience Institute, Hospital Kuala Lumpur, Kuala Lumpur, Malaysia; 2 Department of Neurosciences, School of Medical Sciences, Universiti Sains Malaysia, Health Campus, Kelantan, Malaysia

**Keywords:** superior cerebellar artery, aneurysm, revascularisation surgery, bypass surgery, microsurgery

## Abstract

**Background:**

Superior Cerebellar Artery (SCA) aneurysms account for about 1% to 3% of all intracranial aneurysms. SCA has an interesting anatomy, with multiple options of surgical approaches.

**Methods:**

From January 2022 to May 2023, a total of 167 aneurysm cases were treated at our centre. Only two patients were confirmed to have SCA aneurysm from cerebral angiography. Demographic study, clinical characteristics, radiological data, treatment plan and outcome were analysed.

**Results:**

The total number of vascular cases at our centre from January 2022 to May 2023 is 241. There were 167 cases of aneurysm (69.3%), where 115 patients (68.9%) went for surgery, and 52 (31.1%) opted for endovascular intervention. The distribution of aneurysms by location are ACA 7.2%, ACOM 24.6%, MCA 8.9%, PCOM 18.6%, ICA 21.6%, SCA 1.2%, PCA 1.8%, PICA 8.4%, vertebral artery 2.4%, and basilar artery 5.3%. Complex aneurysms made up 58.7% of all cases and were treated with endovascular procedures more often than simple aneurysms (36.7% vs. 23.2%, *P* = 0.04). Most patients in both the surgical and endovascular groups had good recovery outcomes (79.1% and 76.9%, respectively; *P* = 0.78). Both patients with SCA aneurysms were managed surgically. Two sample cases are presented: i) a proximal saccular SCA aneurysm treated with pterional craniotomy, transylvian approach, and clip reconstruction; and ii) a mid-SCA fusiform, multilobulated aneurysm managed with temporal craniotomy, subtemporal approach, Superficial Temporal Artery (STA)–SCA bypass, and excision of the aneurysm.

**Conclusion:**

SCA aneurysms are rare and present significant surgical challenges due to their complex anatomy. Choosing the right surgical approach depends on the aneurysm’s location along the SCA segments. With proper technique and surgical experience, microsurgical treatment can offer favourable outcomes for patients.

## Introduction

Posterior circulation intracranial aneurysms are uncommon compared to those in the anterior circulation. The most common sites of posterior circulation aneurysm, according to order, are the basilar artery (BA), posterior cerebral artery (PCA), superior cerebellar artery (SCA), and posterior inferior cerebellar artery (PICA). The vertebral artery (VA) and anterior inferior cerebellar artery (AICA) are relatively rare (1). SCA aneurysms are rare, accounting for 1% to 3% of brain aneurysms. Notably, about 28% to 68% of them are usually associated with multiple aneurysms (2–5). They most commonly present with subarachnoid haemorrhage and signs of compression of cranial nerve III, IV or V (3). Interestingly, SCA aneurysms often present with haemorrhage when the size is less than 7 mm (3, 6). This highlights the importance of treating

SCA aneurysms, even when they are small in size. SCA can be divided into four segments: i) the anterior pontomesencephalic (pontine) segment; ii) the lateral pontomesencephalic (ambiens) segment; iii) the cerebellomesencephalic (quadrigeminal) segment; and iv) the cortical segment (3, 6, 7). Patra et al. (4) classified SCA aneurysms into three types: i) Type A – arising from the BA near the origin of the SCA but not involving the SCA; ii) Type B – arising from the SCA–BA junction with involvement of the SCA; and iii) Type C – arising from the distal segment of the SCA (4). SCA aneurysms most commonly arise from the proximal SCA (6, 7). This article reviews and discusses the microsurgical approaches for SCA aneurysms.

## Methods

**Figure 1:**
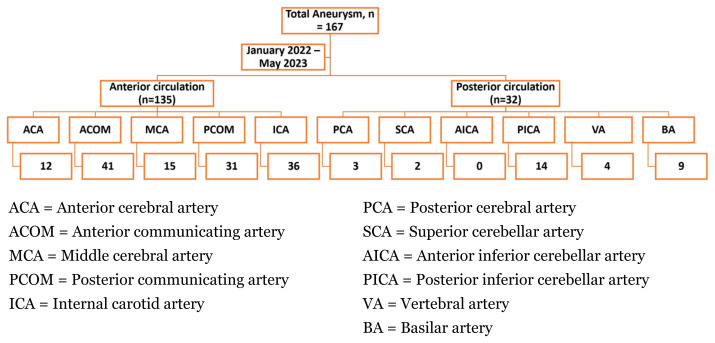
Aneurysmal cases treated with microsurgical and endovascular intervention

This was a retrospective study conducted at a single centre, where the most complex aneurysm cases nationwide are referred for further management. Between January 2022 and May 2023, a total of 167 aneurysm cases were treated at the centre by either microsurgery or endovascular interventions (Figure 1). Only two patients were confirmed to have SCA aneurysm based on cerebral digital subtraction angiography (DSA) or cerebral CT angiography (CTA) findings. Demographic data, clinical characteristics, radiological findings, treatment plans, and outcomes were analysed.

SCA can be divided into four segments: S_1_ – anterior pontomesencephalic (pontine) segment; S_2_ – lateral pontomesencephalic (ambient) segment; S_3_ – cerebellomesencephalic (quadrigeminal) segment; and S_4_ – cortical segment. These are grouped into proximal (S_1_ segment), mid (S_2_ segment), and distal (S_3_ and S_4_ segments). SCA aneurysms are complex when they fulfil the criteria, such as large or giant size, fusiform or blister morphology, multilobulation, intraluminal thrombosis, previous coiling, calcification, branch or perforating arteries arising from the aneurysm, or the presence of multiple aneurysms.

Craniotomies were performed according to the most suitable approach for the different SCA segments. A pterional craniotomy with a transylvian approach was used for a proximal SCA aneurysm, and a temporal craniotomy with a subtemporal approach was employed for a mid-SCA aneurysm. After surgery, the patients were admitted to the neurosurgical intensive care unit for postoperative care. Titanium Alpha clips (Yasargil aneurysm clips) were used for the microsurgical treatments. The suture used for revascularisation, when needed, is DAFILON^®^ 10-0 or 9-0 non-absorbable polyamide monofilament 3/8-circle round-bodied needle. As a standard procedure, for all vascular cases, the vascular mini-Doppler and fluorescein sodium dye are used to assess the complete occlusion of an aneurysm or bypass patency. Therefore, an integrated microscope capable of visualising fluorescein dye within the wavelength range of 560 nm to 690 nm (ZEISS, Kinevo 900) was used.

Outcomes were divided into radiological and clinical outcomes. Clinical outcomes were measured using the modified Rankin Scale (mRS), with scores of mRS 0 to 3 considered good and scores of mRS 4 to 6 considered poor. Radiological outcomes were measured using postoperative CT brain imaging to identify signs of infarction, and angiographic studies (CTA/DSA) to find complete aneurysm occlusions or bypass patency in cases where revascularisation surgery was performed.

### Data Analysis

Descriptive statistics were used to summarise the characteristics of all aneurysm cases. Frequencies and percentages were calculated for categorical variables, including aneurysm complexity, intervention type, and clinical outcome. Comparative analyses were performed using the Chi-square test for categorical variables. Two main comparisons were performed: i) aneurysm complexity versus intervention type – to assess whether complex aneurysms were more likely to be treated surgically or endovascularly; and ii) intervention type versus clinical outcome – to examine whether outcomes differed between surgical and endovascular treatment groups.

All statistical analyses were performed using IBM SPSS Statistics (version 27), and a *P*-value < 0.05 was considered statistically significant. For the rare SCA aneurysm subset, only descriptive statistics were performed due to the small sample size (*n* = 2).

## Results

**Figure 2:**
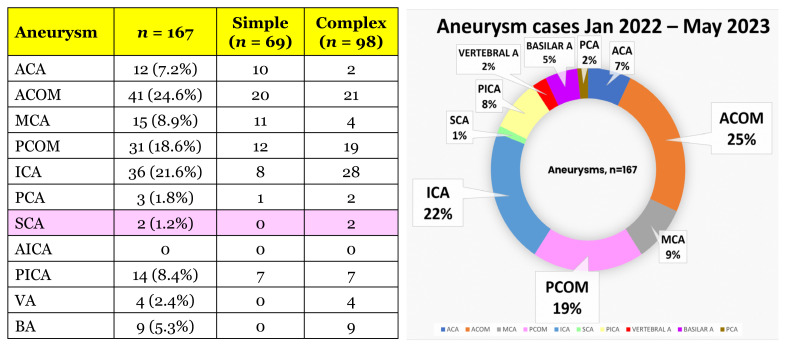
Distribution of aneurysmal cases at the centre

A total of 241 vascular cases were treated at the centre between January 2022 and May 2023 (Table 1). Of these cases, 167 were aneurysms (69.3%). Among them, 115 patients (68.9%) underwent surgery, and 52 patients (31.1%) received endovascular interventions. The distribution of aneurysms according to location is as follows: ACA 7.2%, ACOM 24.6%, MCA 8.9%, PCOM 18.6%, ICA 21.6%, SCA 1.2%, PCA 1.8%, PICA 8.4%, VA 2.4% and BA 5.3% (Figure 2). Both the SCA aneurysm cases underwent surgical intervention – proximal and mid-SCA aneurysm (Table 2). Based on the cases and literature reviewed, approaches to SCA aneurysms were divided into segments, with consideration of their proximity to the BA (Figure 15).

Two representative cases are presented: i) a proximal SCA saccular aneurysm managed with pterional craniotomy, transylvian approach, and clip reconstruction; and ii) a mid-SCA fusiform, multilobulated aneurysm treated with temporal craniotomy, subtemporal approach, Superficial Temporal Artery (STA)– SCA bypass, and excision of aneurysm. The outcome was favourable in the mid-SCA aneurysm case treated with bypass surgery. The most common complication is temporal lobe contusional injury, while other complications include hydrocephalus, cerebellar infarct, and pneumonia.

### SCA Aneurysm Subset

Among the 167 aneurysm cases, two patients (1.2% of aneurysms) were identified with SCA aneurysms. Both were female, aged 44.5 and 53.0 years (mean age 48.8 years). The lesions were complex and located on the left SCA.

The first case involved a ruptured proximal S_1_ saccular aneurysm (4.8 × 6.7 mm), treated via pterional transylvian craniotomy with clip reconstruction. The postoperative course was complicated by contusional and retractional injury, hydrocephalus, lateral cerebellar infarction, and pneumonia, yielding a poor outcome (Glasgow Coma Scale [GCS] E1VTM4, mRS 5). The second case was a triple fusiform SCA aneurysm (S_2_ segments) with associated right cervical ICA fibromuscular dysplasia. The patient underwent subtemporal STA–SCA bypass and aneurysm excision, with a good radiological outcome (GCS E4VTM5, mRS 4). The summary of the SCA cases is shown in Table 2.

### Characteristics of Overall Aneurysm Cases

Of the 167 aneurysm cases, 98 (58.7%) were classified as complex and 69 (41.3%) as simple. In this cohort, complex aneurysms were managed surgically in 63.2% of cases and endovascularly in 36.7%. Meanwhile, simple aneurysms were treated by surgery (76.8%), with endovascular intervention performed in 23.2%.

In terms of clinical outcomes, 131 patients (78.4%) achieved good recovery, 5 (3.0%) experienced guarded outcomes, and 31 (18.6%) had poor outcomes. Among patients who underwent surgical intervention, 79.1% achieved good outcomes, while 76.9% of patients who underwent endovascular intervention had similarly favourable results. The proportions of poor outcomes were similar between the two groups (18.3% for surgery versus 19.2% for endovascular intervention).

### Association between Aneurysm Complexity vs. Intervention and Intervention vs. Outcome

A comparison between aneurysm complexity and intervention showed a statistically significant association (χ^2^ [1, *N* = 167] = 4.29, *P* = 0.028). Although surgery was the main intervention for most of the aneurysm cases, neurosurgeons chose endovascular procedures more often for complex aneurysms (36.7%) than for simple ones (21.7%). This pattern suggests that aneurysm complexity influenced intervention selection, with endovascular methods preferred for more difficult or high-risk cases.

When comparing clinical outcomes between interventions, no major differences were observed between surgery and endovascular treatments. Most patients in both groups achieved good functional outcomes at discharge (79.1% in the surgical group and 76.9% in the endovascular group), and this difference was not statistically significant (χ^2^ [2, *N* = 167] = 1.94, *P* = 0.463). Overall, these findings suggest that while aneurysm complexity may affect the choice of intervention, both approaches provide similar short-term recovery outcomes. The comparisons of aneurysm complexity versus intervention and of intervention versus outcome are summarised in Table 3.

## Discussion

### Case Presentations

This paper discusses the surgical management of SCA aneurysms, with examples of cases and a review of the literature.

### Case 1: Left Proximal SCA Aneurysm

**Figure 3:**
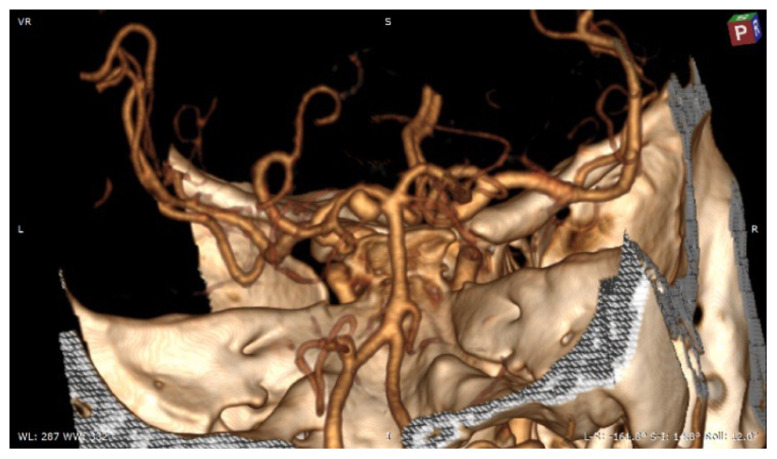
CTA 3D reconstruction shows proximal left SCA aneurysm measuring 4.8 × 6.7 mm

**Figure 4:**
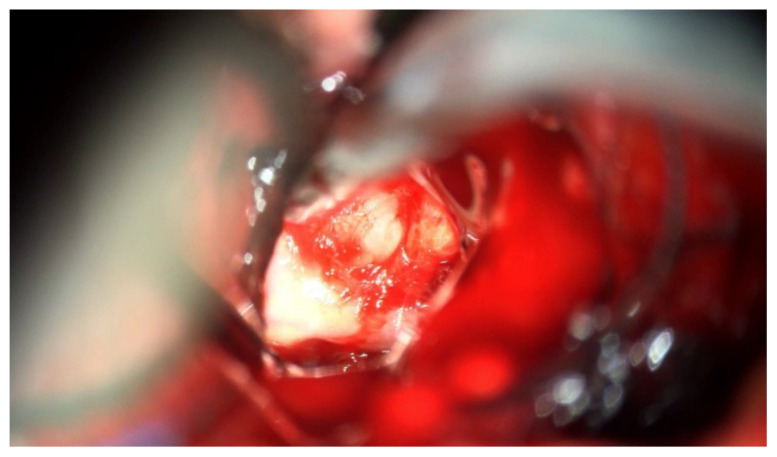
Basilar trunk and proximal SCA aneurysm

**Figure 5:**
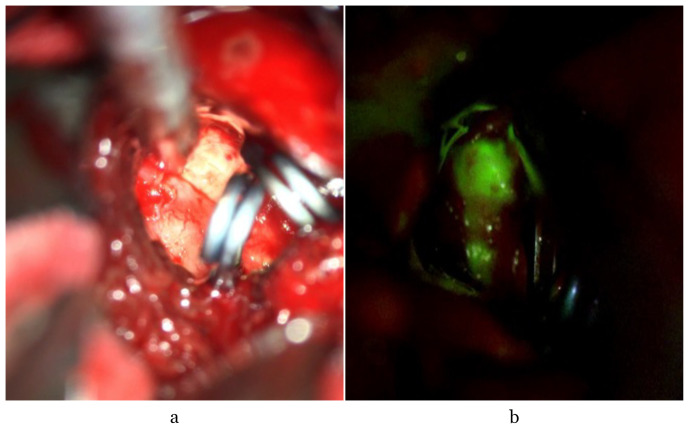
Two titanium clips applied and fluorescein dye given to check total occlusion of the aneurysm: a) Microscopic view of the aneurysm following clip reconstruction; b) Assessment of total aneurysm occlusion and parent artery patency using fluorescein dye

A 51-year-old female with underlying hypertension presented with severe headache and reduced level of consciousness. On admission, her GCS score was E3V4M5, and she was hypertensive. CT brain showed diffuse, thick subarachnoid haemorrhage, especially over the left interpeduncular and crural cisterns. Her World Federation of Neurosurgical Societies (WFNS) grade was 4, and her Fischer grade was 3. Cerebral CTA revealed a proximal left SCA aneurysm measuring 4.8 × 6.7 mm (Figure 3). The patient was scheduled for a left pterional craniotomy, transsylvian approach, and clip reconstruction. A curvilinear incision was made, extending from the widow’s peak to the front of the tragus, followed by a pterional craniotomy. An extradural clinoidectomy was performed for better exposure and visualisation of intradural structures later. The Sylvian fissure was carefully dissected using vascular microinstruments. The MCA, ICA, and PCOM were identified, followed by the basilar trunk, PCA and the SCA aneurysm (Figure 4). Temporary clips were placed at the basilar trunk and contralateral SCA. Subsequently, two Yasargil clips were applied to the aneurysm (Figure 5a).

After clip reconstruction, mini-Doppler and fluorescein dyes were used to assess complete aneurysm occlusion (Figure 5b). Postoperatively, the patient initially recovered well; however, she developed neurogenic pulmonary oedema, a small lateral cerebellar infarct, and severe acute respiratory distress syndrome, which led to significant deterioration. She required a tracheostomy but was later able to wean off the ventilator.

### Case 2: Mid-SCA Fusiform Multilobulated Aneurysm

**Figure 6:**
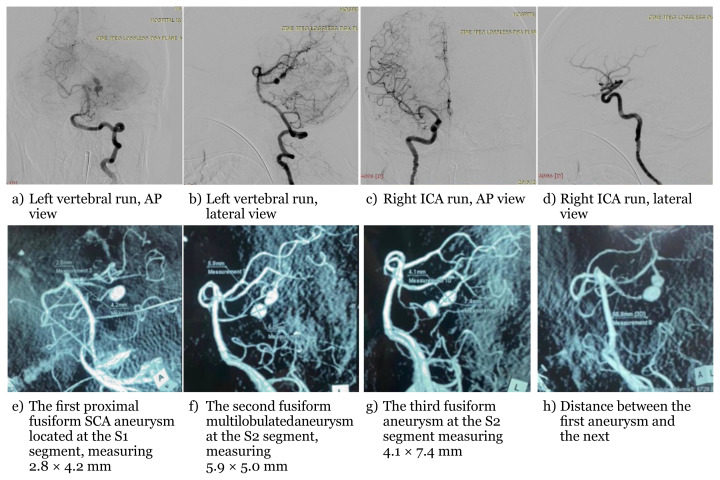
**(a–h)**. Three fusiform multilobulated left SCA aneurysms and right cervical ICA with a beaded appearance, suggesting fibromuscular dysplasia

A 44-year-old Malay female with underlying hypertension presented at the emergency department with severe headache, vomiting and reduced level of consciousness. She was intubated due to low GCS for airway and cerebral protection. CT brain showed diffuse, thick subarachnoid haemorrhage (SAH) with intraventricular haemorrhage and hydrocephalus. Her WFNS and Fischer grades were both 4. Urgent external ventricular drainage was performed, and her level of consciousness improved. Brain CTA revealed two fusiform aneurysms of the left SCA. She was immediately sent to another neurosurgical centre for cerebral DSA and further intervention. Her cerebral DSA showed three fusiform aneurysms of the left SCA (Figure 6).

The first aneurysm was a small fusiform aneurysm located just after its origin at the BA, measuring about 2.8 × 4.2 mm. The second aneurysm was located about 5.5 cm from the first aneurysm, measuring 5.0 × 5.9 mm, and the third aneurysm was just next to the second aneurysm, measuring 4.1 × 7.4 mm; both were fusiform and multilobulated in morphology. The first aneurysm was located at the S_1_ segment, while the second and third aneurysms were located next to each other at the S_2_ segment. In addition, a beaded appearance of the right cervical ICA was noted, raising suspicion of cervical fibromuscular dysplasia.

**Figure 7:**
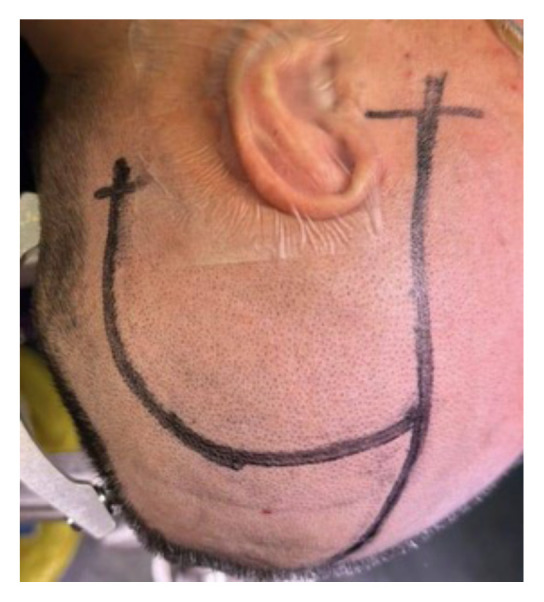
Reverse H shape incision

**Figure 8:**
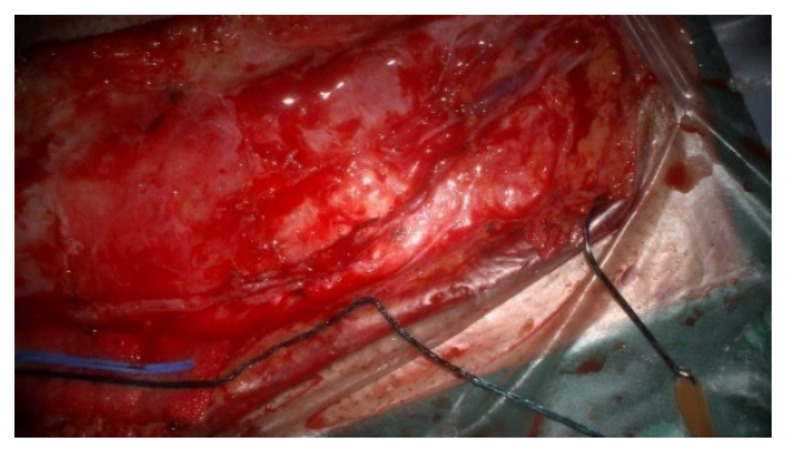
Harvesting the donor artery from the STA

The patient was scheduled for a left temporal craniotomy via a subtemporal approach, with STA-S_2_ SCA bypass and aneurysm trapping. A reverse H-shaped incision was made (Figure 7). The donor artery was harvested first, which yielded about 12 cm of STA (Figure 8). Subsequently, the muscle was elevated, and a basal temporal craniotomy was performed. A C-shaped durotomy was made, followed by gentle retraction of the temporal lobe until adequate exposure of the tentorium and perimesencephalic cistern was achieved. The trochlear nerve was identified, and the tentorium was cut in front of the attachment of the trochlear nerve at the tentorial edge.

**Figure 9:**
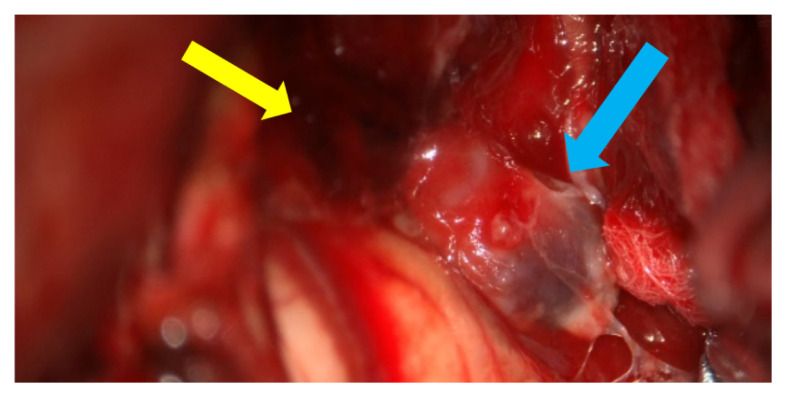
Second multilobulated fusiform aneurysm (with haematoma on top – yellow arrow) and third fusiform aneurysm next to each other at S_2_ SCA (blue arrow)

**Figure 10:**
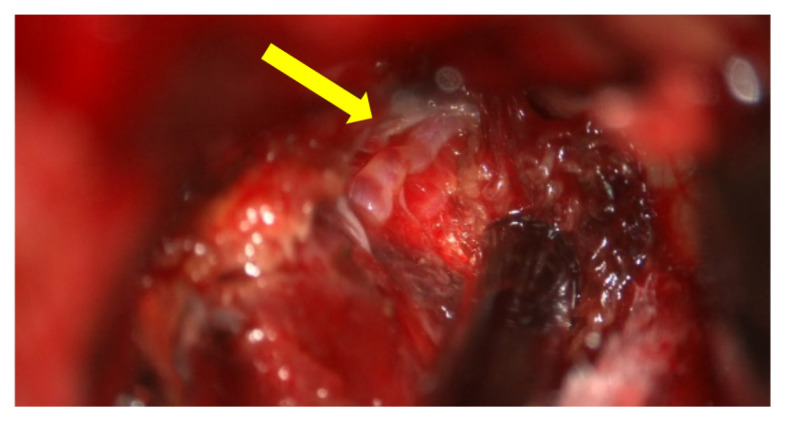
Beaded appearance vessel (yellow arrow) after the first/proximal S_1_ SCA fusiform aneurysm a b

**Figure 11:**
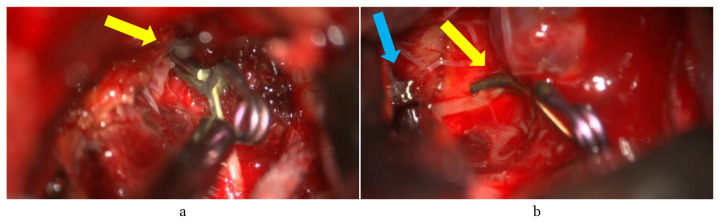
a) Application of proximal clip at proximal S_1_ (yellow arrow); b) Application of distal clip (trapping of aneurysm) (yellow arrow) and clip at recipient artery, distal S_2_ (blue arrow)

**Figure 12:**
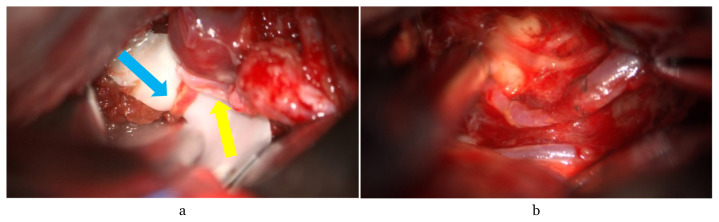
a) Preparation for coaptation of donor, STA (yellow arrow) and recipient artery, S_2_ SCA (blue arrow); b) STA-S_2_ SCA anastomosis using DAFILON® 10-0 non-absorbable polyamide monofilament suture with a 3/8-circle round-bodied needle

After washing out the SAH, the anterior pontomesencephalic, lateral pontomesencephalic, and cerebellomesencephalic segments of the SCA were dissected. All three aneurysms were observed, with a clot noted over the second SCA aneurysm (Figure 9). After the first fusiform S_1_ SCA aneurysm, a beaded appearance of the vessel was seen before reaching the second and third fusiform aneurysms (Figure 10). The revascularisation surgery was performed under propofol burst suppression. A segment of artery distal to the aneurysm, without perforator branches, was identified, dissected, and a temporary clip was applied at the recipient segment (Figures 11a and 11b). An end-to-end anastomosis between the STA and S_2_ SCA was performed, followed by excision of the aneurysm (Figures 12a and 12b).

**Figure 13:**
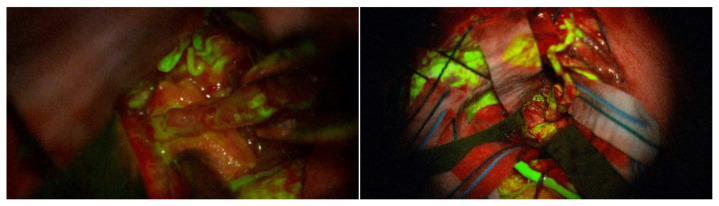
Intraoperative evaluation of bypass using fluorescein dye

**Figure 14:**
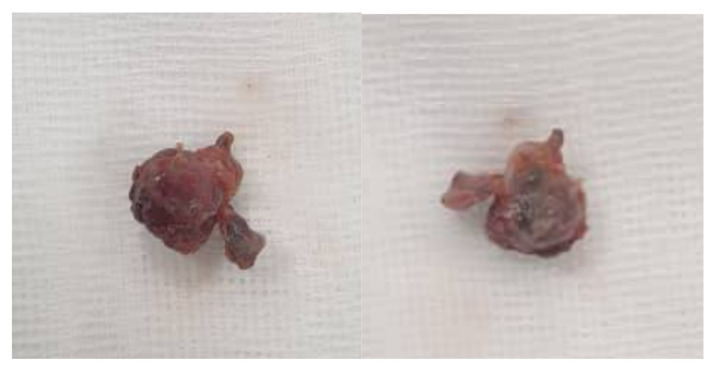
Part of the aneurysm that was excised

The STA that was previously harvested was prepared and instilled with heparin before anastomosis. A “bed-and-pillows” of Gelfoam soaked with papaverine was placed underneath and around the anastomotic field. End-to-end anastomosis was performed using DAFILON^®^ 10-0 non-absorbable polyamide monofilament suture with a 3/8-circle round-bodied needle, and the procedure was completed within 40 minutes. Temporary clips were removed at the end of the procedure, and patency of the anastomosis was confirmed by vascular micro- Doppler and fluorescein sodium dye (Figure 13), as dynamic blood flow measurements were not available at the centre. Finally, the SCA was occluded proximal to the aneurysm, and the aneurysm was excised (Figure 14).

Histopathological examination (HPE) of the aneurysm shows dissection with the presence of revascularisation, areas of fibrosis and disruption of the internal elastic lamina, and increased myxoid accumulation within the media, consistent with features of fibromuscular dysplasia. Postoperatively, the patient was started on antiplatelet therapy to ensure graft patency and for the treatment of fibromuscular dysplasia.

There are many points that need to be considered before deciding on the treatment of SCA aneurysm. Preoperative clinical status, comorbidity, age, and clinical grade are some of the factors to be considered when deciding on surgery. Importantly, if there is a presence of cranial nerve compression or an associated clot, microsurgery is the treatment of choice (4). Some of the morphological factors that need to be considered for surgery of SCA aneurysms include the segment involved, the height of the basilar bifurcation in relation to the posterior clinoid or dorsum sellae, the direction of dome projection, the relation of the SCA to the tentorial edge, the relationship of the SCA to the oculomotor nerve, the configuration of the aneurysm neck and its relation to perforating vessels, the overall complexity, and the orientation of the aneurysm (3, 6). Both patients were young to middle-aged women, with aneurysm domes projecting anteriorly or laterally. The basilar bifurcation was slightly above the posterior clinoid, and a thick SAH/IVH clot was present. Aneurysms are complex in morphology.

**Figure 15:**
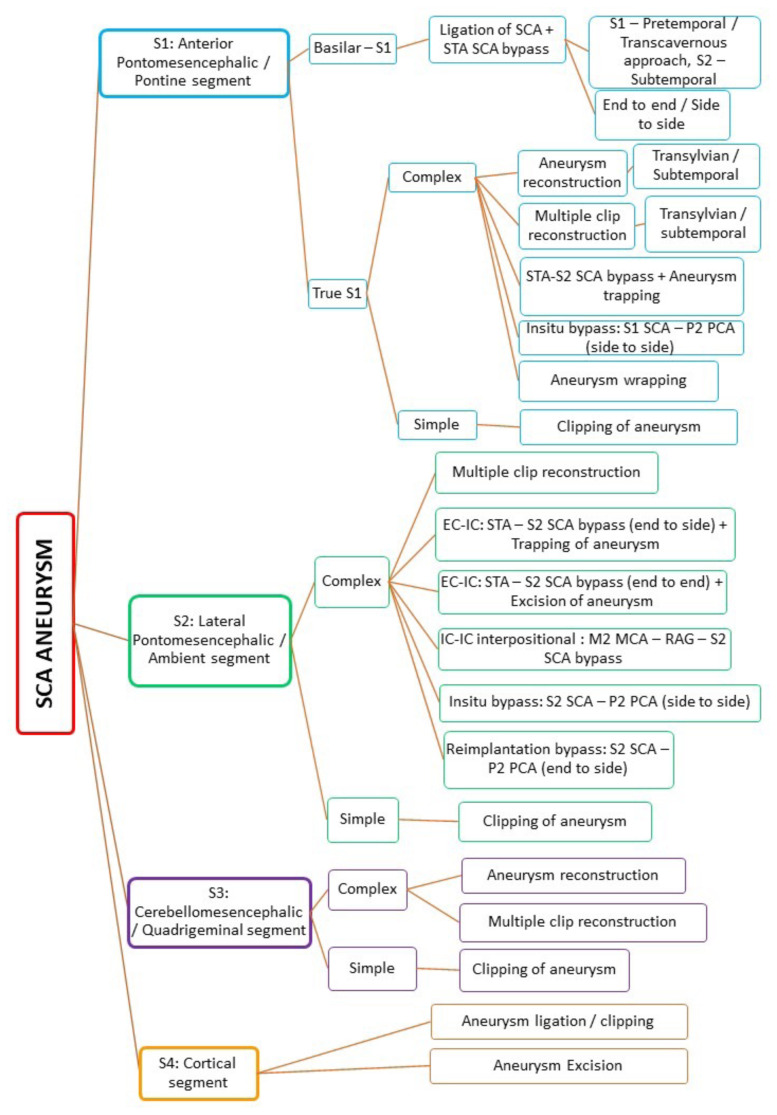
Microsurgical management for SCA aneurysm

Owing to the unique anatomy and location of the SCA, multiple operative approaches and exposures are utilised for different segments (Figure 15 and Table 4). Reported surgical approaches include the lateral supraorbital, subtemporal, occipital, suboccipital and presigmoid approaches, each selected according to the specific SCA aneurysm characteristics (1, 8). For example, a pterional or orbitozygomatic craniotomy can be performed when the SCA aneurysm arises at the SCA origin above the dorsum sellae. However, if the SCA aneurysm is low-lying or below the level of the dorsum sellae, a transcavernous approach gives better exposure, with the help of anterior and posterior clinoidectomy and removal of the upper dorsum sellae. In addition, a retrosigmoid craniotomy gives good exposure for aneurysms located at the anterior part of the mesencephalic fissure and satisfactory exposure when the aneurysm is located at the lateral pontomesencephalic segment. The occipital transtentorial approach gives suitable exposure when the aneurysm is located at the posterior part of the ambient cistern (8).

On the other hand, aneurysms located at the cortical segment can be approached through either a presigmoid or suboccipital craniotomy (3, 6). In the first case, a pterional craniotomy with a transsylvian approach was selected because the aneurysm was located at the proximal segment. For the second case, the subtemporal approach was considered the best option, as revascularisation surgery was planned. Another simple way of planning the operative approach to SCA aneurysms has been described by Patra et al. (4), where the transylvian approach is recommended for type A and B aneurysms, while the subtemporal approach is preferred for type C aneurysms.

Spetzler has suggested a surgical approach for SCA aneurysm according to the involved segments: i) S_1_ segment – pterional or orbitozygomatic or temporopolar; ii) S_2_ segment – temporopolar or subtemporal, with or without anterior petrosectomy or retrosigmoid; iii) S_3_ segment – retrosigmoid or supracerebellar infratentorial; and iv) S_4_ segment – supracerebellar infratentorial or occipital transtentorial (9).

In this study, the surgical approach was divided according to segments, with an additional consideration of aneurysm complexity, as shown in Figure 15. When bypass is needed, especially in complex aneurysms where SCA sacrifice may be needed, STA–SCA bypass is most commonly chosen. This is because it is much more familiar and simpler to most neurosurgeons compared with other types of bypass. STA is considered the best donor artery for bypass because of its similar calibre to the SCA and its ability to give good length when harvested properly. The harvesting site is also easily accessible and familiar to most neurosurgeons. The mean diameter of SCA is around 1.28 mm to 2 mm, which is comparable to STA (1.5 mm to 1.9 mm). The mean length of the anterior segment (S_1_) is around 9.23 ± 4.14 mm, the lateral segment (S_2_) is 11 ± 9.05 mm, and the cerebellomesencephalic segment (S_3_) is 8.6 ± 8.91 mm (2).

Multiple revascularisation techniques are available for posterior circulation aneurysms that require the sacrifice of the parent artery. STA or occipital artery is typically selected as the donor for small vessels (2 mm or less) with a low blood flow rate of 20 to 40 ml/min. When a higher blood flow rate is required (vessels more than 3 mm), radial artery grafts or saphenous vein grafts (flow capacity of ~40 to 70 ml/min) are used (10). When bypass is performed at the S_2_ segment of the SCA, which is conventionally performed, a subtemporal approach is used. In contrast, if the bypass is required at the S_1_ SCA segment, a pretemporal transcavernous approach is performed (8).

Although SCA aneurysms are not common, when compared with other posterior circulation aneurysms such as BA aneurysms, microsurgical treatment of SCA aneurysms gives more favourable outcomes and is associated with lower rates of permanent morbidity and mortality (5, 7). This is probably because SCA is relatively resistant to occlusion. Jin et al. (7) reported that aneurysms at the S_2_–S_3_ segments treated with complete trapping using coils resulted in no neurological deficit, probably due to collateral blood flow and the scarcity of perforators from the S_1_ and S_2_ segments, which reduces the risk of morbid infarction. Another characteristic of SCA aneurysms favouring microsurgical treatment is that the SCA aneurysm neck is often positioned squarely in the carotid-oculomotor triangle, tends to project laterally off the midline, allows a straight line of visualisation, and is usually associated with relatively few perforating arteries (5, 7). Compared with the basilar apex aneurysms, the thalamoperforators located behind the aneurysm are sometimes difficult to visualise and can be easily clipped or injured accidentally during surgery, which may result in infarction. However, such complications rarely develop in SCA aneurysm surgery (5, 7).

The proximal part of the SCA and the BA–SCA junction are classically described as a perforator-free zone, giving surgeons a favourable site of approach. However, there are several studies that reported the presence of perforators in this area, but fewer (around three) compared to those at the basilar tip. When present, these perforating arteries give off branches to the pons before the origin of the lateral branch (3, 4).

Literature reviews have also reported that microsurgery has a higher rate of complete occlusion compared with endovascular treatment. Although many studies have reported better clinical outcomes with endovascular than with microsurgery (88.1% vs. 76.9%), microsurgery gives better radiological outcomes in terms of occlusion rate (90.1% vs. 67.4%) and lower recurrence rate (0% vs. 11.8%) (4). Therefore, it is crucial for neurosurgeons to recognise the importance of microsurgical treatment in posterior circulation aneurysms, especially those involving the SCA.

### Limitations

As a referral centre for complex cases, a large number of challenging aneurysm cases are encountered compared with simple ones. However, rare aneurysms like SCA, PICA, VA and PCA are uncommonly seen, with AICA aneurysms being even rarer. The rarity of these aneurysms makes it challenging to pool them within one centre. Additionally, the number of cases is limited as this is the only centre that performs surgery for complex vascular cases. Thus, a significant analysis of the outcomes could not be accurately evaluated. In addition, the limited number of cases available limits the chances for neurosurgeons, especially the younger ones, to gain more experience and improve complex surgical techniques. Few neurosurgeons are well-trained in complex neurovascular surgery. A more extensive study with a prolonged period of data collection is required in the future. Moreover, serving as the referral centre of difficult cases requires advanced skills that must be acquired and strengthened through local and international training. As a healthcare provider in a developing country, financial constraints affect not only the provision of treatment – including equipment and adjuncts for surgery – but also the availability of training programmes to acquire skills and knowledge. Certain competencies cannot be obtained locally and must be acquired by learning from neurosurgeons abroad.

## Conclusion

SCA aneurysms have been quite an interesting aneurysm to most neurovascular surgeons due to their distinctive location and anatomical characteristics. Their management demands a high-level microsurgical technique and an outstanding understanding of the anatomy. However, microsurgical treatment is often favourable due to its natural qualities and features. SCA aneurysms, which differ from basilar apex aneurysms, have distinct characteristics, including a paucity of perforators, abundant collateral supplies and off-midline location, accompanied by relatively low permanent morbidity and mortality. It also has a higher occlusion rate and lower recurrence rate.

In view of the rarity of occurrence and anatomical uniqueness, different surgical approaches are needed for different segments. The complexity of the surgery changed at different segments, making it more exciting for vascular neurosurgeons. In determining outcomes, not only are surgical skills, facilities and equipment important, but careful patient selection and choice of approach also play a role. The general condition of the patient in the preoperative period and the clinical grade upon presentation are key players in predicting outcome as well.

## Figures and Tables

**Table 1:** Neurovascular cases, *n*(%)

**Total vascular cases January 2022 to May 2023**				241 (100.0)
**Total aneurysm cases**				167 (69.3)
**Total non-aneurysm cases**				74 (30.7)

**Aneurysm cases**				

**Intervention**				

Surgery	115 (68.9)			
Endovascular	52 (31.1)			

**Complexity**			**Surgery**	**Endovascular**

Simple	69 (41.3)		53 (76.8)	16 (23.2)
Complex	98 (58.7)		62 (63.2)	36 (36.7)

**General outcome**			**Surgery**	**Endovascular**

Good	131 (78.4)		91 (69.5)	40 (30.5)
Guarded	5 (3.0)		2 (40.0)	3 (60.0)
Poor	31 (19.3)		21 (67.7)	10 (32.3)

**Outcome according to intervention**		**Good**	**Guarded**	**Poor**

Surgery	115 (68.9)	91 (79.1)	2 (1.7)	21 (18.3)
Endovascular	52 (31.1)	40 (76.9)	3 (5.8)	10 (19.2)

**Table 2:** SCA aneurysm cases (*n* = 2)

**Case**	1	2
**Age (years)/sex**	53/F	44.5/F
**Aneurysm type**	Saccular	Fusiform multilobulated (triple aneurysm)
**Location / segment/ side**	Proximal / S1 / anterior pontomesencephalic (pontine) / left	Mid / S2 / lateral pontomesencephalic (ambiens) / left
**Size (mm)**	4.8 × 6.7	2.8 × 4.2; 5.0 × 5.9; 4.1 × 7.4
**Intervention**	Left pterional craniotomy, transylvian *approach, and clip reconstruction	Left temporal craniotomy, subtemporal approach, STA–SCA bypass and excision
**Complexity**	Complex	Complex
**Duration of surgery (hours)**	2	5
**Interval to definitive treatment (days)**	4	4
**Pre-op neurological status**	E3V4M5	E1V1M2
**Complications**	Postoperative contusion, retractional injury, hydrocephalus, cerebellar infarct, and severe pneumonia	Small temporal retraction contusional injury
**Radiological outcome**	Poor	Good
**Outcome after surgery (GCS/mRS)**	E1VTM4 / 5	E4VTM5 / 4

**Table 3:** Comparison of aneurysm complexity versus intervention and intervention versus mRS outcome

Category	*P*-value (Chi-square test)	Interpretation
Complexity vs. intervention	0.028	Significant difference
Intervention vs. mRS outcome	0.463	No significant difference

**Table 4:** Microsurgical management for SCA aneurysm

Approaches	
**S** **_1_** ** segment:**	**S** **_3_** ** segment:**
Pterional or orbitozygomatic craniotomy, Pretemporal transcavernous	Retrosigmoid craniotomy
Pterional or orbitozygomatic craniotomy, Transylvian	Suboccipital craniotomy, supracerebellar infratentorial
Temporal craniotomy, subtemporal ± Transylvian	**S** **_4_** ** segment:**
**S** **_2_** ** segment:**	Suboccipital cranitomy, supracerebellar infratentorial
Temporal craniotomy, subtemporal ± anterior petrosectomy	Occipital craniotomy, transtentorial
Retrosigmoid craniotomy	Presigmoid, posterior transpetrosal

## References

[1] You W, Meng J, Yang X, Zhang J, Jiang G, Yan Z (2022). Microsurgical management of posterior circulation aneurysms: a retrospective study on epidemiology, outcomes, and surgical approaches. Brain Sci.

[2] Dodevski A, Tosovska Lazarova D, Zhivadinovik J, Lazareska M, Stojovska-Jovanovska E (2015). Morphological characteristics of the superior cerebellar artery. Pril Makedon Akad Nauk Umet Odd Med Nauki.

[3] Arraez MA, Dominguez M, Sanchez-Viguera C, Ros B, Ibañez G, July J, Wahjoepramono E (2019). Surgery of superior cerebellar artery aneurysm (SCA). Neurovascular surgery.

[4] Patra DP, Bir SC, Maiti TK, Kalakoti P, Cuellar-Saenz HH, Guthikonda B (2016). Superior cerebellar artery aneurysms, the “sui generis” in posterior circulation: the role of microsurgery in the endovascular era. World Neurosurg.

[5] Rodríguez-Hernández A, Walcott BP, Birk H, Lawton MT (2017). The superior cerebellar artery aneurysm: a posterior circulation aneurysm with favorable microsurgical outcomes. Neurosurgery.

[6] Nair P, Panikar D, Nair AP, Sundar S, Ayiramuthu P, Thomas A (2015). Microsurgical management of aneurysms of the superior cerebellar artery – lessons learnt: an experience of 14 consecutive cases and review of the literature. Asian J Neurosurg.

[7] Jin SC, Park ES, Kwon DH, Ahn JS, Kwun BD, Kim CJ (2012). Endovascular and microsurgical treatment of superior cerebellar artery aneurysms. J Cerebrovasc Endovasc Neurosurg.

[8] Rodríguez-Hernández A, Rhoton AL, Lawton MT (2011). Segmental anatomy of cerebellar arteries: a proposed nomenclature. Laboratory investigation. J Neurosurg.

[9] Russin JJ, Spetzler RF, Spetzler RF (2015). Microsurgical management of aneurysms of the posterior cerebral, superior cerebellar, and anterior inferior cerebellar arteries. Neurovascular surgery.

[10] Evans JJ, Sekhar LN, Rak R, Stimac D (2004). Bypass grafting and revascularization in the management of posterior circulation aneurysms. Neurosurgery.

